# Optimization of physical conditions for the production of thermostable T1 lipase in *Pichia guilliermondii* strain SO using response surface methodology

**DOI:** 10.1186/s12896-017-0397-7

**Published:** 2017-11-10

**Authors:** Mary Ladidi Abu, Hisham Mohd Nooh, Siti Nurbaya Oslan, Abu Bakar Salleh

**Affiliations:** 1 Enzyme and Microbial Technology Research Center, Universiti Putra Malaysia, 43400 Serdang, Selangor Malaysia; 2 Department of Biochemistry, Faculty of Biotechnology and Biomolecular Sciences, Universiti Putra Malaysia, 43400 Serdang, Selangor Malaysia; 3 Institute of Bioscience, Universiti Putra Malaysia, 43400 Serdang, Selangor Malaysia; 4 Department of Biochemistry, Faculty of Applied and Natural Sciences, Ibrahim Badamasi Babangida University Lapai, Niger State, Minna, Nigeria

**Keywords:** Box-Behnken design, Plackett-Burman design, Physical condition, *Pichia guilliermondii*, Thermostable T1 lipase

## Abstract

**Background:**

*Pichia guilliermondii* was found capable of expressing the recombinant thermostable lipase without methanol under the control of methanol dependent alcohol oxidase 1 promoter (AOXp 1). In this study, statistical approaches were employed for the screening and optimisation of physical conditions for T1 lipase production in *P. guilliermondii*.

**Result:**

The screening of six physical conditions by Plackett-Burman Design has identified pH, inoculum size and incubation time as exerting significant effects on lipase production. These three conditions were further optimised using, Box-Behnken Design of Response Surface Methodology, which predicted an optimum medium comprising pH 6, 24 h incubation time and 2% inoculum size. T1 lipase activity of 2.0 U/mL was produced with a biomass of OD_600_ 23.0.

**Conclusion:**

The process of using RSM for optimisation yielded a 3-fold increase of T1 lipase over medium before optimisation. Therefore, this result has proven that T1 lipase can be produced at a higher yield in *P. guilliermondii*.

## Background

Lipases (triacylglycerol acylhydrolases) are natural catalysts, catalysing the hydrolysis of long chain triacylglyerides into their simpler di- and monoacylglycerol, as well as glycerol forms [1, 2]. Lipases are abundantly found in nature originated from plants, animals and microbes [3]. Meanwhile, microbial thermostable lipases are known to be originated from bacterial, fungi and yeast with many applications in industrial and biotechnological processes [4–6].

Some lipase producing yeasts belonged to *Pichia, Kluyveromyces*, *Candida* and *Torulaspora* genre are methylotrophic in nature [7–9]. They metabolise methanol as their carbon and energy source [10–12]. Recombinant protein expression in methylotrophic yeasts are driven by the strong regulatory AOXp 1. Furthermore, activation of AOXp 1 for gene expression and protein production in some methylotrophs is independent of induction by methanol, even in the presence of AOXp 1, which is a mechanism referred to as de-repression and methanol induced expression [10, 13, 14] . Conventionally, biomass generation and gene expression phases are distinct to each other The carbon source used is either glycerol or glucose, which is mostly repressive to gene expression as it vary from each yeast and is a determining factor on the level of biomass generated [15]. Yurimoto and co-authors illustrated that the level of AOXp 1 activities in different carbon sources is related to their gene expression [10]. In some yeasts it is known as methanol induced expression while in some, it is a de-repressed activation of gene expression depending on the carbon source, level of de-repression and choice of host [12, 16].

However, a new yeast *P. guilliermondii* isolated from spoilt orange was used as a recombinant host for the expression of thermostable T1 lipase gene isolated from *Geobacillus zalihae* [17, 18]. Similar to methylotrophic yeasts, gene expression and protein production in *P. guilliermondii* have been found to be driven by methanol induced AOXp 1 [18]. In this study, *P. guilliermondii* was found capable of generating biomass using glycerol as a carbon source and relatively undergoing gene expression without any carbon source as observed in the study on *Hansenula polymorpha* [10] depending on the level of de-repression.

Previously, *Escherichia coli* has been used as a recombinant host for T1 lipase production, but was characterised by low expression [19]. It is important to note that T1 lipase gene has been also cloned and expressed in *P. pastoris* but recently, it was observed that *P. guilliermondii* was observed as a better producer of T1 lipase under the control of AOXp 1 [18]. Therefore, it is appropriate to screen and optimise the enzyme production, initially by media optimisation, followed by optimisation of physical conditions significant for the enhancement of T1 lipase production in *P. guilliermondii*.

Varying one parameter at a time (Conventional method) in a bid to optimise production process is labour intensive, time consuming and ignores the co-interaction between parameters, leading to errors in identifying the optimum production conditions [20, 21]. As such, recent studies have shown the use of statistical approaches, which eliminates the limitation associated with the use of the conventional method of optimisation. Plackett-Burman Design (PBD) has been used in several biotechnology applications for screening multiple parameters and identifying the best parameters with significant effects [22]. Box-Behnken Design (BBD) optimisation method has been also employed for effective optimisation of PBD experiments [23]. However, there is no known study made on the optimisation of physical conditions during the production of recombinant T1 lipase. This gap in knowledge could affect the accurate and effective production of T1 lipase in *P. guilliermondii*. Thus, this study aimed at optimising buffered and non-buffered media as well as physical conditions for the production of recombinant T1 lipase using PBD and BBD Response Surface Methodology.

## Materials and methods

### Strain and inoculum preparation

The recombinant yeast *P. guilliermondii* from previous study [17] was maintained on YPD- Zeocin agar plate (1% yeast, 2% peptone, 2% Bacteriological agar, 20% dextrose and 100 μg/mg Zeocin). A single colony was inoculated into 10 mL YPD broth at 30 °C with an agitation of 250 rpm overnight.

### Cultivation media

Two different media were used for T1 lipase production, which are buffered and non-buffered media (BMY and YPT, respectively). BMGY medium (Buffered complex glycerol medium - 1% yeast extract, 2% peptone, 1% 100 mM potassium phosphate pH 6.0, 1.34% yeast nitrogen broth, 0.4 mg/ L biotin and 1% glycerol), and YPTG (Yeast extract, Peptone, Tryptic Soy Broth and Glycerol) were used for biomass generation. Meanwhile, BMY (Buffered complex medium) with composition similar to BMGY without glycerol and YPT with composition similar to YPTG without glycerol were used as lipase production media.

### Determination of lipase activity.

Lipase activity was assayed colorimetrically [24] with slight modifications [25]. 2.5 mL olive oil, 50 mM phosphate buffer (pH 9) emulsion (1:1, *v*/v) and 20 μl of 0.02 M CaCl_2_ were added to 1 mL of culture filtrate. The reaction mixture was incubated for 30 min at 70 °C. Enzyme reaction emulsion was terminated by adding 1 mL of 6 N HCl and 5 mL of isooctane. The reaction mixture was vortexed for 30 s and two layers were formed. 4 mL of the upper isooctane layer containing the fatty acid was transferred into a new test tube for analysis after that 1 mL copper (II) pyridine reagent (pH 6.1) was added and vortexed for 30 s. The absorbance of the upper layer was read at 715 nm. One unit of lipase activity was defined as the rate of free fatty acid formed in μmole per minute.

### Plackett-Burman (PB) screening design

Factorial designs were evaluated at levels −1 and +1 for low and upper level respectively. The parameters screened are inoculum size, incubation time, temperature, pH, culture volume and agitation speed. Twelve sets of trial experiments were generated from Design Expert (version 7.0.) software package. PBD was patterned in line with the first-order model (*Y* = *Σβ*0 + *Σβ*p*Xp*), where, Y is the response (T1 lipase), β0 is the intercept, β is the coefficient of the variable and Xp is the independent variable. Screened parameters were represented on Pareto chart of standardised effects.

### Box-Behnken design (BBD)

Physical parameters were evaluated at three different interactive levels, which are low (−), middle (0) and high levels (+). Statistical design was used for optimisation involving 17 sets of trial experiment, to determine the co-efficient of the model, as well as to predict the response and its experimental validation. The response was fitted into a second order polynomial equation (Eq. 1) and optimum levels were represented in response surface plots.

1 $$ \boldsymbol{\mathsf{Y}}=\boldsymbol{\mathsf{\boldsymbol{\varSigma}\boldsymbol{\beta }}}\mathbf{0}+\boldsymbol{\varSigma} \boldsymbol{\beta} \boldsymbol{pXp}+\boldsymbol{\mathsf{\boldsymbol{\varSigma}\boldsymbol{\beta }qXq}}+\boldsymbol{\mathsf{\boldsymbol{\varSigma}\boldsymbol{\beta }zXz}}+\boldsymbol{\mathsf{\boldsymbol{\varSigma}\boldsymbol{\beta }p}}\mathbf{2}\boldsymbol{\mathsf{Xp}}\mathbf{2}+\boldsymbol{\mathsf{\boldsymbol{\varSigma}\boldsymbol{\beta }q}}\mathsf{2}\boldsymbol{\mathsf{Xq}}\mathbf{2}+\boldsymbol{\mathsf{\boldsymbol{\varSigma}\boldsymbol{\beta }z}}\mathsf{2}\boldsymbol{\mathsf{Xz}}\mathbf{2}+\boldsymbol{\mathsf{\boldsymbol{\varSigma}\boldsymbol{\beta }pqXpq}}+\boldsymbol{\mathsf{\boldsymbol{\varSigma}\boldsymbol{\beta }pzXpz}}+\boldsymbol{\mathsf{\boldsymbol{\varSigma}\boldsymbol{\beta }qzXqz}}+\boldsymbol{\epsilon} $$

where, Y is the response, β0 the intercept, β the coefficient of the variable, Xp, Xq, Kz are equal to variable (parameter) 1, 2 and 3, Xpqz represent the linear effect, X(p^2^q^2^z^2^) are the quadratic form, X(pq,pz,qz) are the interactive effects and ϵ is the residual relative to the experiment .

### Statistical analysis

Responses observed, were analysed using two way analysis of variance (ANOVA). This analysis was generated having the definition for independent variables experimented. The linear, quadratic and interaction regression coefficient of each term in the model were determined. Using the F-value at a probability (P) of 0.05 and confidence level of 95%, the significance of all terms in the polynomial was statistically analysed and all coefficients were computed using Expert Software version 7.0.

## Results and discussion

### Effects of different media on T1 lipase production

**Fig. 1:**
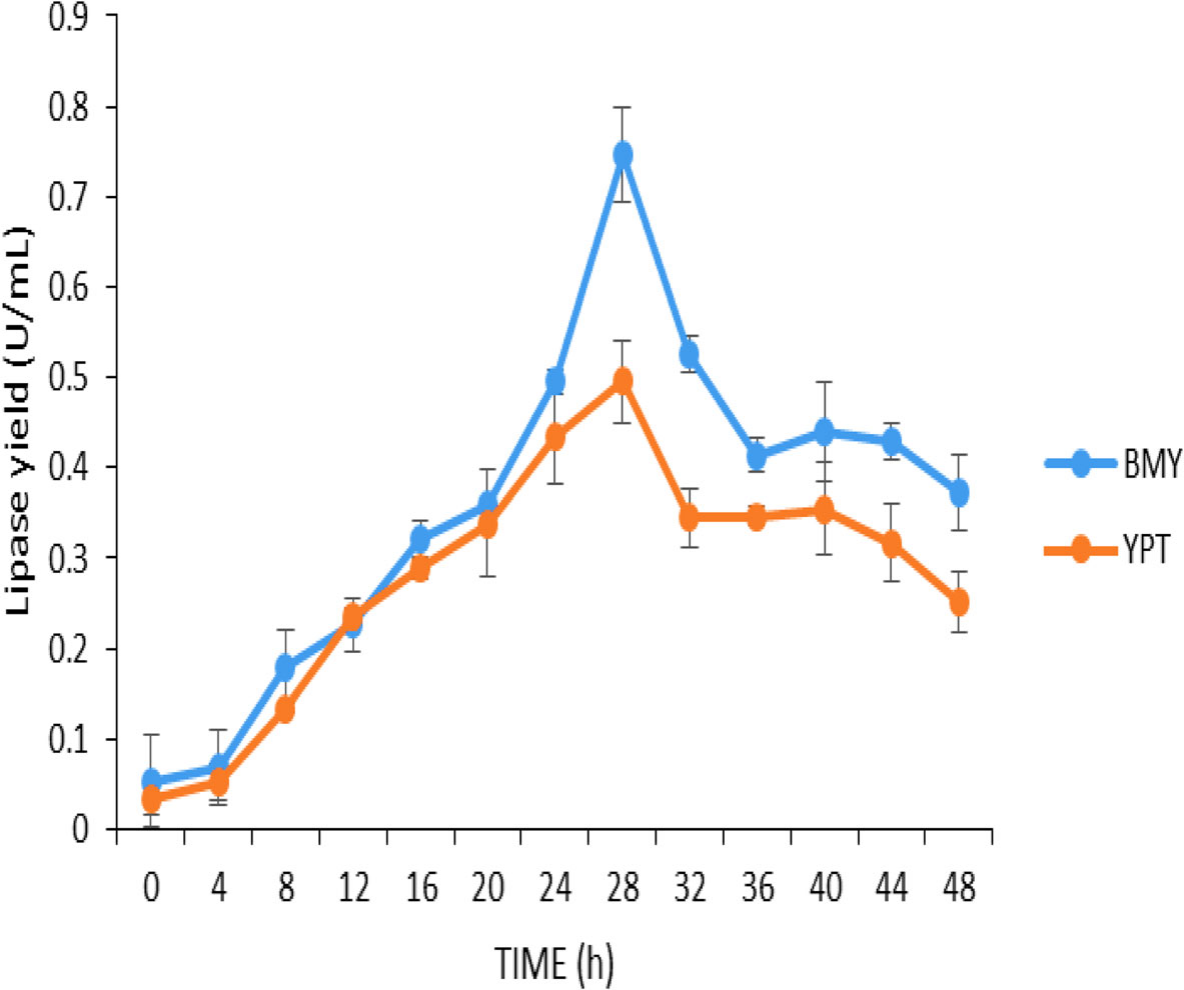
Optimisation of different media for T1 lipase production. This shows the production of T1 lipase in a buffered and non-buffered media for selection of an optimum medium for lipase production. BMY-buffered complex medium (blue line), YPT-yeast extract peptone tryptic soy broth (orange line)

Alterations in the pH of a cultivation medium can lead to a change in the charge of a medium, which affects cell growth and recombinant protein production. pH is mentioned as a crucial fermentation factor affecting cell growth and recombinant proteins production if not controlled [26–28]. In this study, recombinant T1 lipase was produced in a pH controlled medium (BMY) and non-pH controlled medium (YPT). Figure 1 illustrates that T1 lipase production was higher in BMY with T1 lipase yield of about 0.7 U/mL compared to YPT with a yield of 0.5 U/mL. Both media gave optimum production after 28 h of cultivation and declined afterwards. On the other hand, both media provided vitamins and trace elements, as growth enhancers for biomass and energy generation during enzyme production. Furthermore, BMY provided a constant pH condition for T1 lipase production.

### PBD

**Fig. 2:**
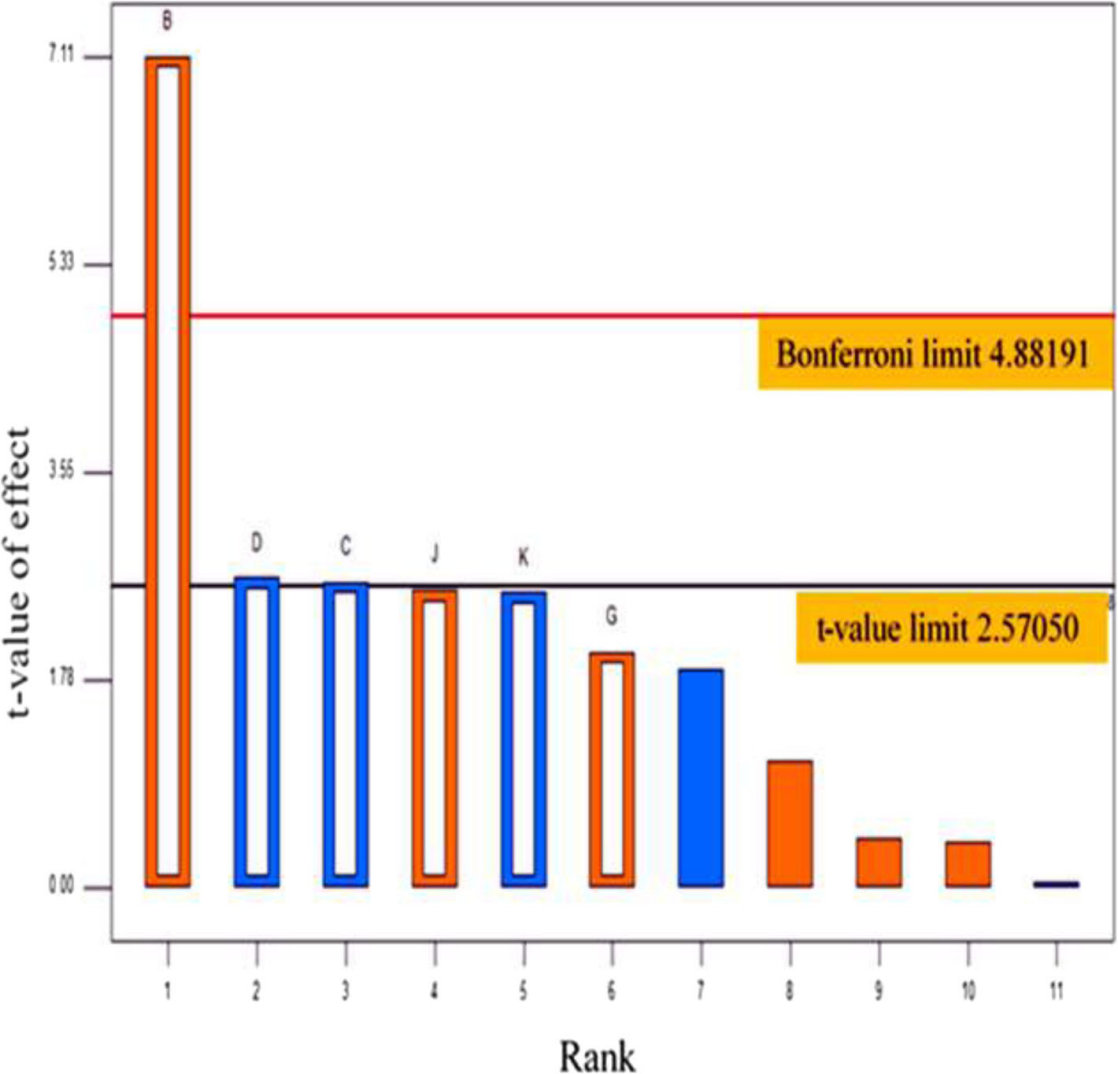
Pareto chart of standardized effects ranking. B-pH, D-incubation time, C-inoculum size, J, K, G-Dummies and the unlabeled bars are the insignificant parameters. Bonferroni limit (pink line)-indicates absolute significance and t-value (black line)-indicates line of significant effect. Blue and orange bars represents parameters screened for significant effect for T1 lipase production. Bars above or on t-value limit line of significance, indicates parameters having positive influence on T1 lipase production

Six parameters were screened, and their effects on T1 lipase production were observed (Table 1). These parameters are pH, incubation time (IT), inoculum size (IS), culture volume (CV), temperature (T) and agitation speed (AS). Using Pareto chart (Fig. 2), significant and non-significant physical parameters were placed above and below the horizontal line (t-value limit) respectively, in descending order. The vertical line shown in the chart indicted the statistical significance (*P* > 0.05), at 95% confidence level. The results obtained for each parameter showed that pH > incubation time > inoculum size, exerted significant effects on T1 lipase production.

**Table 1:** Plackett-Burman screening Design Methodology

Run	Factor 1 A: T (°C)	Factor 2 B: pH	Factor 3 C: IS (%)	Factor 4 D: IT (h)	Factor 5 E: AS (rpm)	Factor 6 F: CV (mL)	Response 1 LA (U/mL)	Response 2 OD_600_ (nm)
1	30.00	6.00	2.00	24.00	300.00	50.00	1.4	16. 5
2	30.00	10.00	2.00	24.00	250.00	200.00	2.5	24.8
3	20.00	6.00	4.00	24.00	300.00	200.00	1.0	17.9
4	20.00	6.00	2.00	48.00	250.00	200.00	1.5	27.9
5	20.00	10.00	4.00	48.00	250.00	50.00	1.3	20.9
6	20.00	10.00	2.00	48.00	300.00	50.00	2.4	23.9
7	20.00	10.00	4.00	24.00	300.00	200.00	2.3	20.2
8	20.00	6.00	2.00	24.00	250.00	50.00	1.5	25.6
9	30.00	10.00	2.00	48.00	300.00	200.00	1.9	21.8
10	30.00	6.00	4.00	48.00	250.00	200.00	1.0	22.0
11	30.00	10.00	4.00	24.00	250.00	50.00	2.4	25.4
12	30.00	6.00	4.00	48.00	300.00	50.00	1.4	22.3

*LA* lipase activity, Factors same as parameters, Response same as observation and OD-Optical Density

### Optimisation of screened parameters using BBD

Parameters identified by PBD experiment (pH, incubation time and inoculum size) having significant effects on T1 lipase production, were further examined and optimised through BBD methodology. This method allows the interaction of pH, incubation time and inoculum size in a 17 trial randomised experiments (Table 2) with their optimum levels observed as maximum amount of lipase was produced.

**Table 2:** Box-Behnken (BB) screening design

RUN	Factor: 1 A:pH	Factor: 2 B: IT (h)	Factor: 3 C: IS (%)	LA (U/mL)	OD_600_ (nm)
1	10	36	4	1.8	24.9
2	10	48	3	0.9	24.4
3	8	36	3	1.2	21.8
4	6	36	2	1.6	22.3
5	8	48	2	0.9	23.9
6	6	48	3	1.0	21.0
7	8	36	3	1.1	23.6
8	8	24	2	1.9	24.6
9	8	48	4	0.7	24.4
10	8	36	3	0.9	26.0
11	6	36	4	1.4	21.4
12	10	24	3	2.1	22.6
13	8	36	3	0.6	21.3
14	8	24	4	1.6	22.8
15	6	24	3	1.8	21.9
16	10	36	2	2.7	22.1
17	8	36	3	1.6	22.3

Run-flask number, *IS* inoculum size, *IT* incubation time, *LA* lipase activity, *OD* optical density

Statistical analysis of variance (Table 3) was used to investigate the effectiveness of BBD model. The F-value (Fisher’s statistical analysis) and the *p*-value (<0.0001) were used as tools for evaluating the significance of the model. The model terms of Prob > F (less than 0.05) were deemed significant while, those with prob. > F greater than 0.10 were considered insignificant [29, 30]. The analysis of BBD model had a prob. > F of 0.0004, which was considered significant to T1 lipase production. The ‘Lack of Fit’ (Table 3), which was insignificant to the model, was considered good.

**Table 3:** BB Statistical Analysis of Variance

Source	Sum of Square	df	Mean Square	F-Value	P-Value Prob > F	
Model	2.40	7	0.34	42.64	0.0004	Significant
Residual	0.040	5	8.033E-003			
Lack of Fit	0.030	4	7.592E-003	0.77	0.6807	Not Significant
Pure Error	9.800E-003	1	9.800E-003			

R^2−^0.9835

Adj R^2−^0.9605

Pred. R^2^–0.8814

Std Dev. - 0.0902

*Pred* Predicted, *Adj* Adjustable R^2^, *Std Dev* Standard deviation

Generally, the coefficient for determination for model variable (R^2^), and its prediction of the response is significant when, its value is closer to 1, which is 100% [31, 32]. Thus, the model was considered significant with over 98% of model variability (R^2^–0.9835) explained. The adj R^2^ with value of 0.9605 was in reasonable agreement with the predictable R^2^–0.8814. The signal to noise ratio was measured by a term called adequate precision. Any ratio greater than 4 is acceptable [32, 33]. In this model, the ratio was19.512, which is greater than 4 thus, it was considered acceptable.

To further explain the dependence of T1 lipase production on physical parameters, a second order polynomial equation (Eq. 2) was generated from the statistical analysis of BBD model.

2 $$ \boldsymbol{Lipase}\boldsymbol{activity}=+{\mathbf{1.13}}^{\ast}\boldsymbol{\mathsf{A}}-{\mathbf{0.43}}^{\ast}\boldsymbol{\mathsf{B}}-{\mathbf{0.2}}^{\ast}\boldsymbol{\mathsf{C}}-{\mathbf{0.029}}^{\ast }{\boldsymbol{\mathsf{A}}}^{\ast}\boldsymbol{\mathsf{C}}+{\mathbf{0.033}}^{\ast}\boldsymbol{\mathsf{C}}+{\mathbf{0.41}}^{\ast}\boldsymbol{\mathsf{A}}\mathsf{2}+{\mathbf{0.16}}^{\ast}\boldsymbol{\mathsf{C}}\mathbf{2} $$

Where A = pH, B = induction time and C = inoculum size.

### Diagnostic case tools

**Fig. 3:**
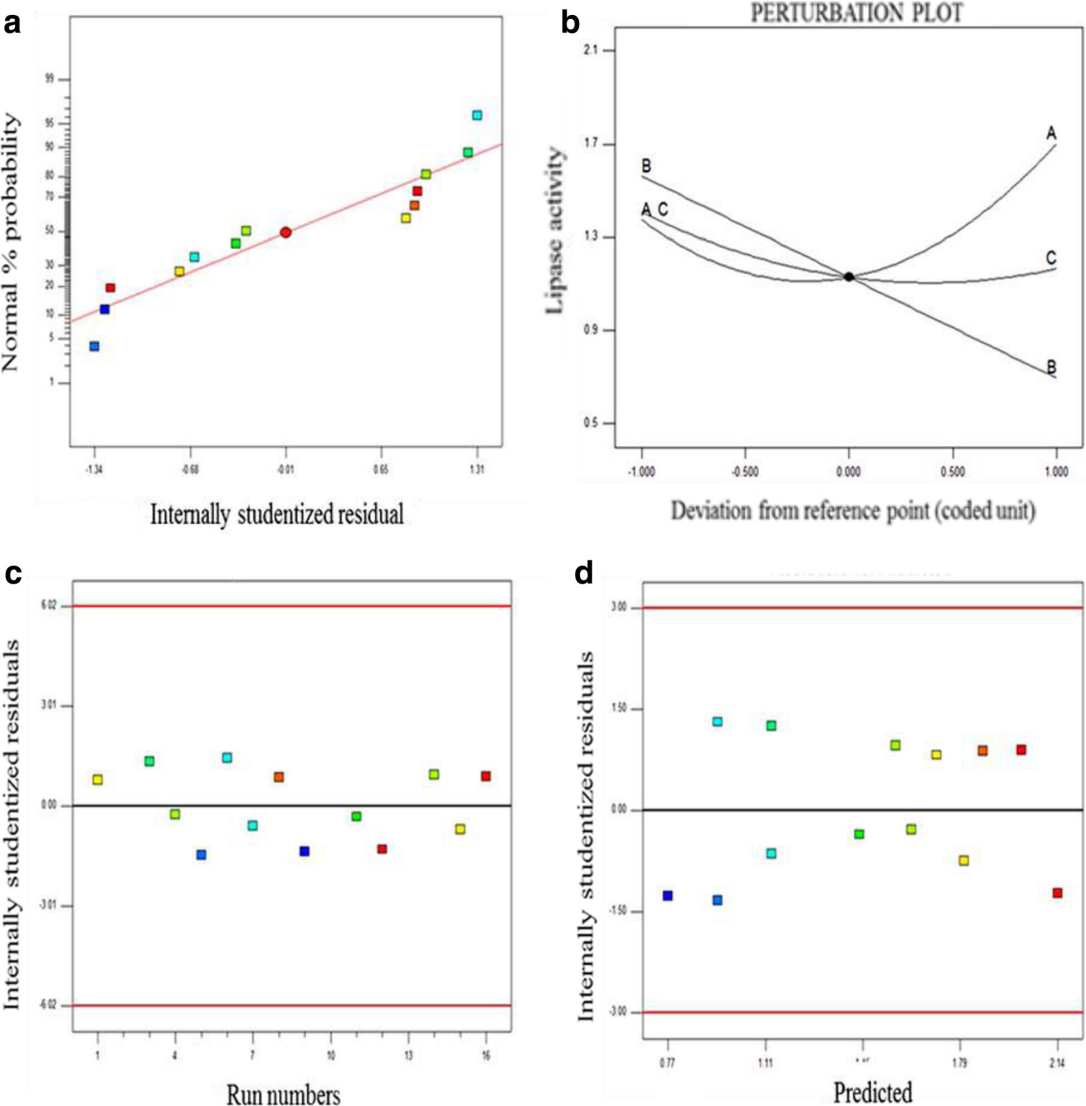
Diagnostic case statistic plots of data points. **a** Is a normality plot, showing studentized residual data points conforming to the assumption of normal percentage probability distribution by aligning themselves along the straight line of regression. **b** Perturbation plot represents significant parameters in form of lines with alphabets at the end of each line. The most significant parameter is the line with the most curve with respect to the reference point at the center. A-pH, B-incubation time, C-inoculum size. **c** are internally studentized residuals for run numbers (experimented data) and **d** internally studentized residuals for predicted data points. Both figures show data points evenly distributed within limit in a straight line with no data point exceeding the red horizontal lines

Normality percentage plot measures standard deviations, adequate prediction and interpretation of the model. Normality percentage plot was used as a tool to check the efficiency of the model and to infer if the internally studentised residuals follows a normal distribution where data points fall along the straight line of regression. This is necessary prior response surface optimisation of the model to avoid misleading results from the model [34, 35]. Values observed (Fig. 3a) aligned close to the ideal line of regression indicated the accuracy and significance of the model, which was in line with the normal distribution assumption. Besides, perturbation plot (Fig. 3b), was used as a comparative tool to determine the sensitivity of individual parameter to the response (T1 lipase). A deviation from the reference point indicated the magnitude of effect exerted by a parameter on the response, which confirmed the efficiency of the model. Parameter ‘A’ (pH) was observed to have the most deviation (curvature) from the reference point. This indicated the sensitivity of the response to pH and the significance of pH to T1 lipase (response) production. Deviation of parameter ‘C’ (inoculum size) from the reference point was reported though the deviation was less intense compared to Parameter ‘A’. The deviation of parameter ‘B’ (induction time) to the reference point was observed to be insignificant to T1 lipase production. Studentised residuals (Fig. 3c and d) are often used to explain the systematic deviation from the hypothesis that errors examined are often independent of each other and evenly distributed [36, 37]. It also measures the values of standard deviation that distinguishes experimental values (actual values) and values generated from BB model design (predicted) experiments [38]. In this study, residual errors were within the allowable limits having an observed random error distribution without confirming any pattern but maintaining a constant variation. The adequacies of these diagnostic plots indicated the applicability of the models produced by RSM for optimally producing T1 lipase in *P. guilliermondii*.

### 3D response surface and contour plots

**Fig. 4:**
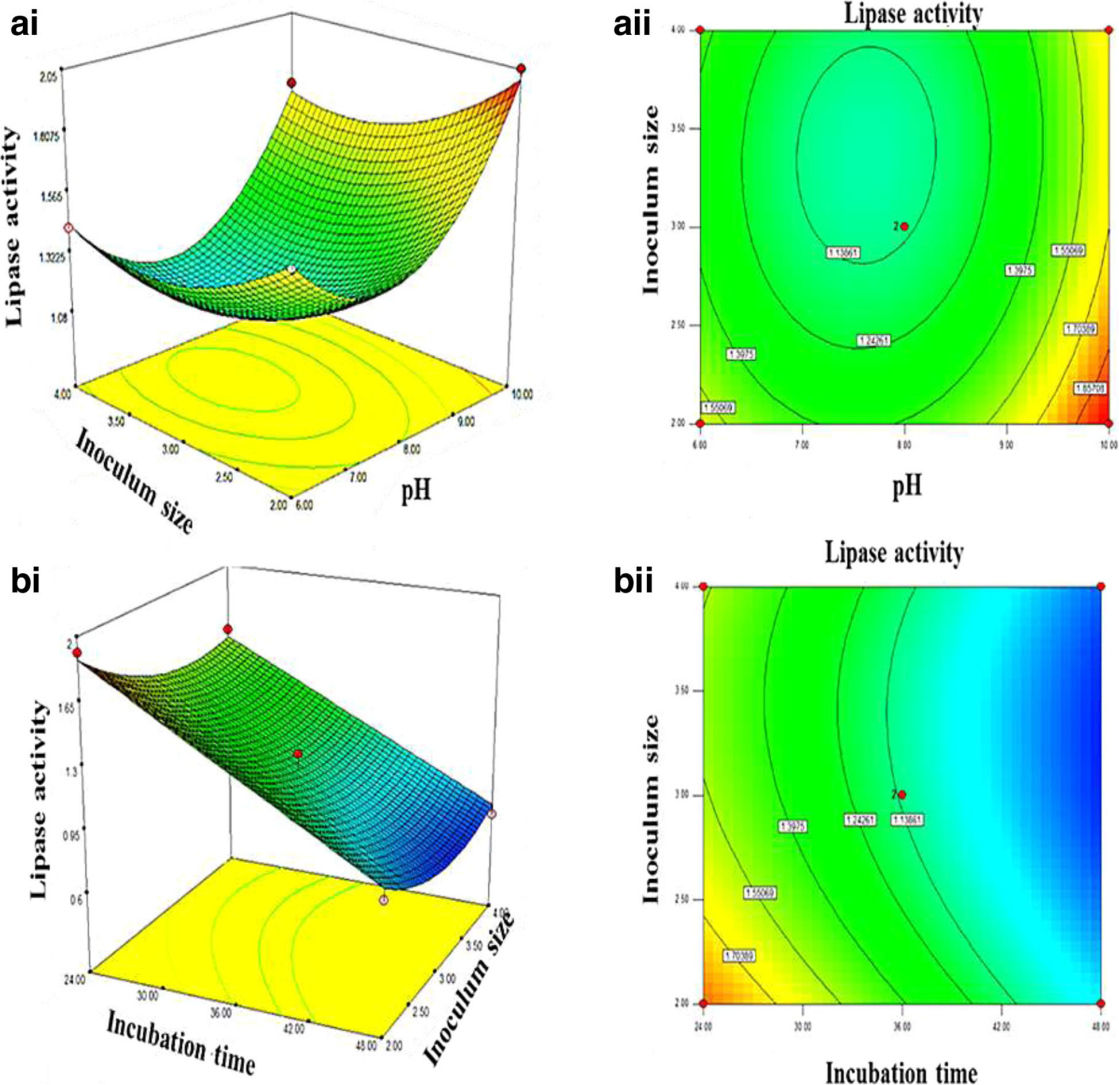
Three dimension response surface plots and contour plots. Shows interactions between two parameters and their effect on T1 lipase production while, the third parameter is kept constant. Elliptical contour (**bi** and **bii**) indicates significant interaction while, circular contour (**ai** and **aii**) signifies negligible interaction

The interaction of two parameters and their effects on lipase production were evaluated while, maintaining the third parameter at a constant level. 3D responses aided in the visual determination of optimum levels of each parameter as they interact. Basically, the 3D responses have either elliptical representation, which is considered significance or the circular representation which is considered to be negligible [39, 40]. As the level of these parameters changes, the interactive effects they exert on the response (recombinant T1 lipase) are also changed. Interaction between pH and inoculum size was studied while, maintaining incubation time at a constant value of 36 h (Fig. 4a). At a very high pH of 10 and inoculum size of 2% lipase production was optimum with a yield of 1.9 U/mL. Decrease in lipase production became trendy as a decrease in pH was observed with subsequent increase in inoculum size. In Fig. 4b, lipase production was found to be optimum in 24 h incubation time, whereas 2% of the inoculum size, pH was maintained at a constant range of eight (8). The level of lipase produced at these optimum parameters levels is 1.7 U/mL.

## Validation of the model

**Fig. 5:**
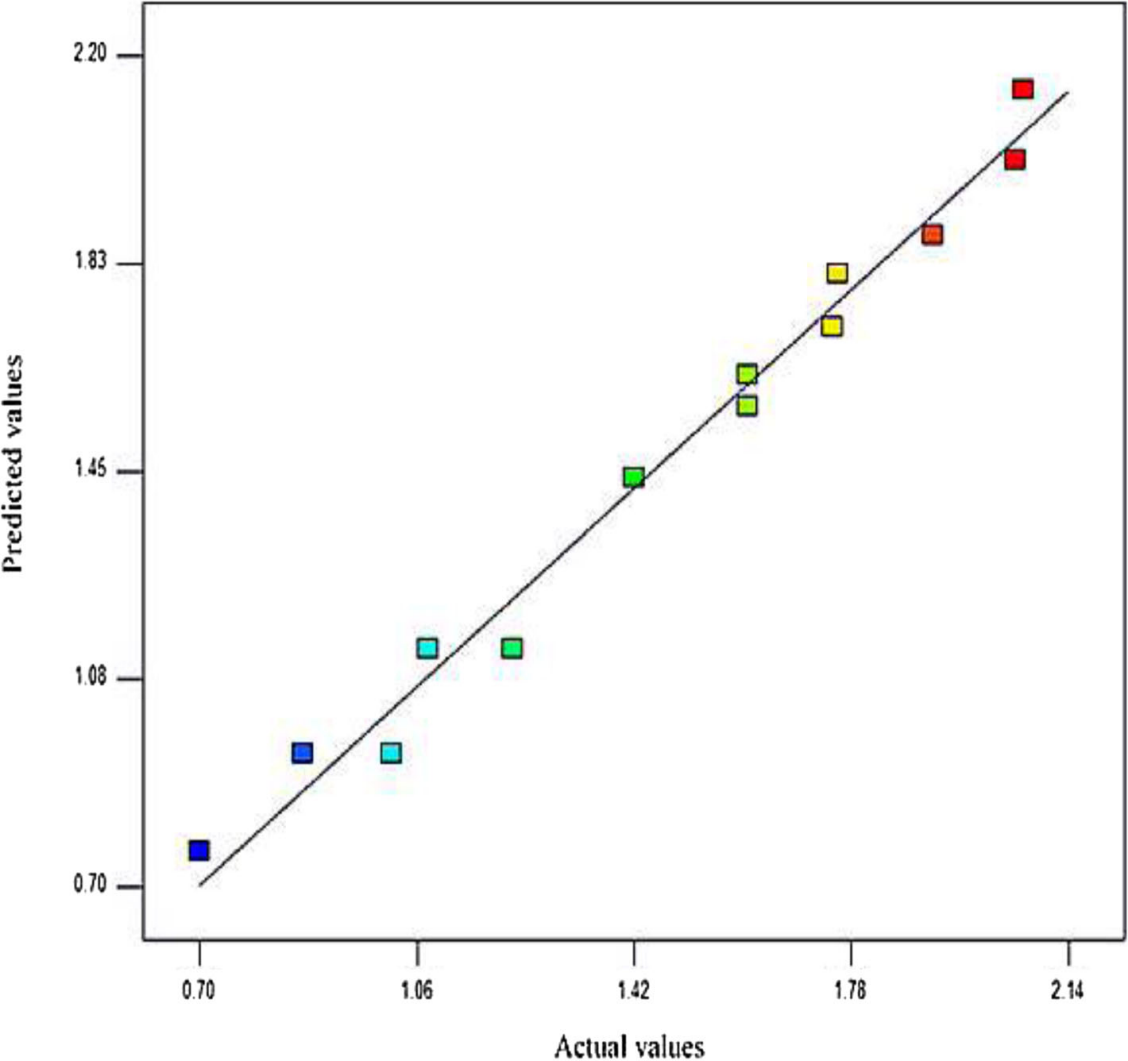
Validation of Predicted versus Actual values. The straight line of regression with data points across indicates the suitability of the model, agreement between predicted and actual values and it conforms to the assumption of data point representation

The accuracy of the model was determined (Fig. 5). A point prediction of the model was generated by the statistical analysis software. pH (6), incubation time (24 h), inoculum size (2%) lipase yield of 2.1 U/mL and biomass of OD_600_ 24.6 were predicted. These predicted parameters were experimented, demonstrating the experimental yield for lipase production of 2.0 U/ mL and a biomass of OD_600_ 23.0. This observed result showed a high degree of correlation with the predicted one. Hence, the model was considered accurate and reliable.

## Conclusion

This study is the first attempt of optimising T1 lipase yield in *P. guilliermondii* under the regulatory control of AOXp 1, without methanol. Statistical methods of PBD and BBD were used to screen and optimise physical conditions (parameters). This methods allowed the elimination of physical conditions that were insignificant to T1 lipase production while, providing a platform to study the individual and combined effects of a reduced number of physical conditions essential to lipase production. It was observed that pH, incubation time and inoculum size were physical conditions significance to T1 lipase production in *P. guilliermondii.* These conditions were optimised at various randomised fermentation levels while the predicted conditions for lipase yield were experimentally validated. The observed relationship between the predicted and experimented values as they aligned in straight line to the line of regression has indicated the suitability of the model in predicting conditions and optimised levels at which lipase production was optimum. A 3-fold increase in lipase yield after optimisation was observed, with 300% increase in T1 lipase production. RSM has been proven to be effective in enhancing T1 lipase production in *P. guilliermondii* without induction by methanol, thus reducing the cost of production and hazards associated with methanol usage.

## Abbreviations

%: Percentage
AG: Agitation speed
BBD: Box-Behnken Design
BMGY: Buffered complex – glycerol medium
BMY: Buffered complex - medium
CaCl_2_: Calcium Chloride
CV: Coefficient of variation
CV: Culture volume
Df: Degree of freedom
h: hour
IS: Inoculum size
IT: Incubation time
L: Liter
M: Molar
mg: Milligram
mL: Milliliter
mM: Millimole
^o^C: Degree Celcius
OD_600_: Optical Density at 600 nm
PBD: Plackett- Burman Design
R^2^: Regression coefficient
Rpm: Rotation per minute
sec: Second
SS: Sum of Square
Std Dev: Standard deviation
v/v: Volume per volume
w/v: Weight per volume
YPD: Yeast extract Peptone Dextrose
YPT: Yeast extract Peptone Tryptic soy
YPTG: Yeast extract Peptone Tryptic soy Glycerol
μg: Microgram
